# Modelling underreported spatio-temporal crime events

**DOI:** 10.1371/journal.pone.0287776

**Published:** 2023-07-12

**Authors:** Álvaro J. Riascos Villegas, Jose Sebastian Ñungo, Lucas Gómez Tobón, Mateo Dulce Rubio, Francisco Gómez

**Affiliations:** 1 Quantil, Bogotá, Colombia; 2 Facultad de Economia, Universidad de los Andes, Bogotá, Colombia; 3 Statistics and Public Policy, Carnegie Mellon University, Pittsburgh, Pennsylvania, United States of America; 4 Facultad de Ciencias, Departamento de Matemáticas, Universidad Nacional de Colombia, Bogotá, Colombia; 5 Laboratorio de Analítica de Datos (DataLab), Universidad Nacional de Colombia, Bogotá, Colombia; Utrecht University: Universiteit Utrecht, NETHERLANDS

## Abstract

Crime observations are one of the principal inputs used by governments for designing citizens’ security strategies. However, crime measurements are obscured by underreporting biases, resulting in the so-called “dark figure of crime”. This work studies the possibility of recovering “true” crime and underreported incident rates over time using sequentially available daily data. For this, a novel underreporting model of spatiotemporal events based on the combinatorial multi-armed bandit framework was proposed. Through extensive simulations, the proposed methodology was validated for identifying the fundamental parameters of the proposed model: the “true” rates of incidence and underreporting of events. Once the proposed model was validated, crime data from a large city, Bogotá (Colombia), was used to estimate the “true” crime and underreporting rates. Our results suggest that this methodology could be used to rapidly estimate the underreporting rates of spatiotemporal events, which is a critical problem in public policy design.

## Introduction

The observation of crime events constitutes a primary input used by government agencies for designing citizens’ security strategies [1, 2]. Different instruments aim to register these observations, including official crime record systems, citizen victimization surveys, and offender self-reports of crimes committed [2]. Nevertheless, the underreporting biases, introduced by unequal crime reporting across social groups and geographical areas [3–6], underrecording tendencies of official entities [7], which commonly prioritize the registration of high-impact offenses, methodological limitations in the selection of victims/offenders representative samples in the case of surveys [8, 9], or simply, the lack of observers to report crime occurrences [8] highly impacts the number and type of offenses known through these mechanisms. Therefore, activities that, by some criteria, are considered crimes may occur without being registered by the systems devised to count them [10]. This phenomenon obscures our knowledge of crime dynamics and is known as the “dark figure of crime” [8].

The dark figure of crime has severe consequences: (1) it limits the deterrent capacity of the criminal justice system, (2) causes victims to become ineligible for public and private benefits, and (3) it affects insurance costs, among others [10]. In addition, in citizen security planning, which requires time-varying and trustable reports of crime incidences for resource allocation [2, 11, 12], heterogeneous dark crime figures in different geographic areas may also result in the misallocation of police resources, hampering short-time planning as demonstrated by [9, 13]. These geographic variations result from complex victim underreporting dynamics and inconsistencies in recording practices across different jurisdictions [14, 15].

Inconsistencies in police practices may add random measurement errors to official crime statistics [15], but heterogeneous systematic negative counts in the form of underreporting affect the conclusions that can be drawn from the data. Such underreporting can be caused by a variety of factors, as documented by [16]. For instance, the severity of the crime, community attributes such as social and organizational networks, and personal characteristics such as demographic attributes, attitudes toward the police, or past experiences with law enforcement, influence the decision to seek help and to report a crime to the authorities. If a region with a high crime rate also has a high rate of underreporting, another region with less crime and less underreporting may seem in the crime statistics as a more insecure region and receive more security resources than the former. Furthermore, the dark figure of crime obscures the crime dynamics theories formulated and tested with official data. Therefore, clarifying crime’s dark figure over time and space constitutes a paramount necessity in security planning.

Different strategies for estimating real crime incidences based on data describing crimes exist [6, 9, 17, 18]. These strategies mainly rely on official crime reports and citizen victimization surveys and their covariates, including demographical and economic costs linked to the crime. Most approaches rely on victimization surveys, originally proposed to provide a ground truth of crime incidence. For instance, using these crime observations, Buil-Gil et al. provide long-term estimations of crime incidence for small areas [9]. Similarly, Akpinar et al. [19], and Buil-Git et al. [18] used surveys to simulate crime occurrences. Because of their design and intention, these victimization surveys provide a closer spatial picture of the criminal dynamic. However, despite the importance of these instruments for highlighting dark crime, they also result in noisy crime observations because of methodological limitations related to the sample design and its limited capability for capturing time-varying crime changes [8]. Victimization surveys may also be affected by underreporting due to fear or the victims forgetting information over time [8, 20]. These crime measurements are also limited in sample size by the available budget [8]. In addition, neither victimization nor repeated victimization is randomly distributed, likely resulting in a sampling bias that may impact underreporting [8, 21].

Alternatively, official crime registers, collected and available over time, provide indirect but time-updated views of the crime dynamics. Therefore, these observations have also been considered to improve the “true” crime characterization [17]. In particular, Gillespie adjusted the number of reported crimes of official statistics with the inverse probability of reporting a crime. This probability resulted from considering the costs of the crime and the benefit of informing it [17]. More recently, Chaudhuri et al. [18], and Moreira et al. [22] assumed crime as a linear function of demographic covariates and accounted for an additional term linked with inefficiency in the citizen’s report, i.e., underreporting. Although these last approaches may provide a short-time estimation of actual crime incidences, they require exogenous covariates, which may also vary in time, limiting their capabilities for underreporting crime estimation over time. In summary, both data sources provide indirect information about the crime. Estimated crime rates provided by victimization surveys provide a closer spatial view of the crime dynamic, while estimations from official criminal records observe temporal crime dynamics. Nevertheless, current approaches still need to be expanded to offer time-varying estimates of crime incidence.

In recent years, several governmental agencies established alternative mechanisms to observe crime-related phenomena, including telephone citizen’s reports [23], in-situ citizen’s field reports [24], and mobile-based reports [25], among others. Currently, these observational mechanisms are deeply integrated into the citizen’s security management information systems [2], registering large amounts of crime-related observations almost online, i.e., crime data is sequentially available during the operation of the information systems [26]. Although these observations still suffer from the dark figure of crime [8], together they may potentially provide valuable information for complementing official records. Recently, different works explored data integration/fusion approaches to provide more information about crime from multiple crime observation sources [27], particularly by spatially combining estimations of crime from different data sources, such as official crime reports, calls to the emergency line related to crime, and citizen’s contraventions [27]. Nevertheless, these approaches are limited in uncovering crime underreporting over time (online) because there are no mechanisms for integrating partial observations arriving online into previous crime observation data.

The main objective of this work was to study the identification over time of the “true” unknown crime incidence rates based on official reports of crime incidents and the “true” underreporting rates based on complementary information, particularly crime-related data acquired gradually over time. In contrast with previous works aimed to describe underreporting in long-time scales by exploiting victimization surveys [6, 9, 19], official crime data [17, 18, 22], or combining multiple crime data sources [27], this work aims to integrate additional incremental evidence about crime once is available, allowing to gain knowledge about the crime phenomena gradually, instead of forcing to wait for final integration. The proposed online estimation relies on a new crime underreporting combinatorial multi-armed bandit model [28] aimed to elicit the “true” average incidence rates and estimate the underreporting rates for different spatial units over time. Importantly, the proposed approach maximizes the number of observed incidents and allows for limited budgets [28]. We hypothesized that the online estimation of underreported crime data, considering partial complementary observations of the “true” crime, might help to estimate underreporting rates over time, further clarifying the dark figure of crime. Historical data of more than 35.000 yearly crime incidents from two instruments for crime observation were used to study this hypothesis: 1) officially reported crimes and 2) telephone citizen reports on crimes in Bogotá (Colombia). The combination of these two data sources provided an approximation to the “true” crime incidents, which was aimed to be discovered by the proposed approach using officially reported crimes and partial observations of the citizens’ reports acquired over time. In addition, the proposed strategy was also explored in the underreporting crime estimation evidenced in victimization surveys [19]. For this, Bogotá’s victimization survey, which reports both victimization and underreporting rates, was used to simulate underreported crime incidents. Then the proposed strategy identified the underreporting crime rates. The main contributions of this work are, first, the introduction of a new model aimed to provide estimates of the “true” crime incident rate over time, and second, the quantitative evaluation of the capacity of the model to unveil underreported crimes for different crime data sources. This work may have implications for designing cost-effective planning mechanisms for citizens’ security planning.

The remainder of this paper is organized as follows. The Materials and Methods section introduces the main ideas of the proposed approach and the formal underreporting model in a multi-armed bandit setting. To solve this problem, we introduce and evaluate three well-known algorithms using simulated data and show how this strategy can elicit the “true” crime incidence and underreporting rates in a large city. Then, simulated results unveiling the dark figure of crime are reported. The Discussion section explains our contribution and provides a general discussion, and the last section concludes with the results of this study.

## Materials and methods

### Proposed model

**Fig 1:**
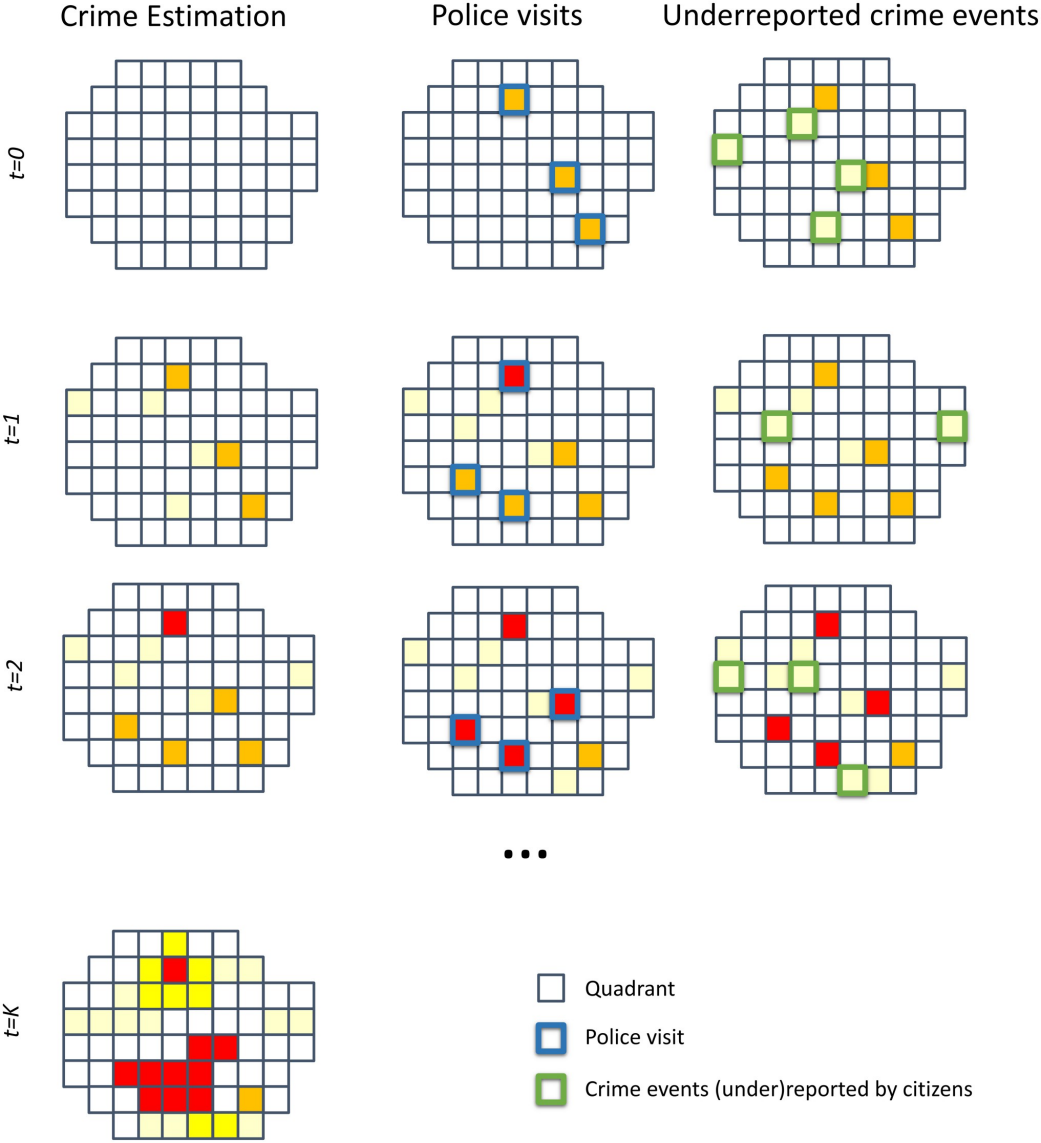
The dark figure of crime estimation. Daily gathering crime observations obtain a daily update for the crime estimation (filled-in colors of small squares). Information coming from police visits (blue border squares), which decision planners can control, updates these estimations. Simultaneously, the information provided by crime events reported by citizens (green border squares) is also integrated. The decision planner may account for exploration-exploitation strategies by dynamically locating police visits.

Fig 1 illustrates the proposed approach. First, let’s assume that the city has a set of quadrants (small squares in Fig 1) with unknown distributions of crime that want to be discovered. Furthermore, each quadrant has its crime distribution with unknown expected values (represented by filled-in colors of small squares in Fig 1). The model aims to estimate the expected value of crime as close to the “real” average of crime by daily gathering crime observations.

For this, suppose these quadrants can be repeatedly visited on daily rounds, for instance, by the police, gathering information about the actual crime occurrences. Blue border squares illustrate these police visits in the second column in Fig 1. In principle, if all quadrants are visited, these “real” crime observations may unveil the dark figure of crime. However, because of budget limitations, only limited subsets of quadrants can be visited daily by the police. For instance, given a fixed budget, Fig 1 shows that only three quadrants can be visited. Consequently, the “real” figure of crime remains hidden because of the limited number of quadrants visited. At the same time that police is visiting a few quadrants, partial crime observations can be gathered to estimate quadrant crime underreporting, for instance, using citizen complaints, as illustrated by the green border squares in the underreported crime events column of Fig 1.

Nevertheless, a planning agent can dynamically assign daily the quadrants to be visited, resulting in an exploration mechanism of the true-crime dynamics. For instance, note how different blue/green border quadrants are selected/reported in different places at other times (rows) in Fig 1. The proposed model aims to provide a strategy to choose the subset quadrants to be visited daily, maximize the number of true crimes observed and receive partial crime occurrences from data collected from citizens’ complaints. With this description, this problem is an instance of what we call a combinatorial multi-armed bandit (CMAB) problem with an underreporting.

The CMAB with an underreporting problem is a class of sequential decision problems in which, at each iteration, a planning agent with a limited budget (for instance, some coins) needs to choose amongst *M* arms or actions (for example, a set of slot machines) to maximize the cumulative reward obtained by those actions (for example, the total money resulting from playing on the slot machines). In the proposed model, the actions correspond to a combinatorial object, precisely, the subset of quadrants to be visited daily for informing the “true” crime. The limited budget refers to the maximum number of quadrants the police may visit (for instance, the number of blue border squares in Fig 1). The reward depends on the crime observations reported from the visited quadrants. Importantly, in this class of sequential problems, the planning agent may also account for partial feedback provided by other arms, not necessarily visited in a round, to help decision-making. In this case, this partial feedback will correspond to the criminal complaints reported by citizens in different quadrants, as illustrated by the third column of Fig 1.

To solve the CMAB with an underreporting problem, i.e., select the quadrants to be visited daily, it is worth observing that the subset of actions not only provides a mechanism for exploration, i.e., monitoring the true crime dynamic at the whole city level, but also for exploitation, i.e., potentially observing more crimes in particular city areas, for instance, by focusing the visits on the quadrants with the highest mean of estimated crime. The exploration is exemplified by Fig 1 at times 0 and 1, where police may visit previously unvisited quadrants highlighting “real” crime. In contrast, the exploitation is illustrated at time 2, where the police visit quadrants with high rewards, i.e., high observed crime. In addition, the principle of optimism in the face of uncertainty, i.e., the more uncertain a quadrant is about crime, the more critical it becomes to explore it, can guide the actions’ location. These two facts are exploited by the so-called combinatorial upper-confidence bound algorithm (CUCB) [29]. This algorithm assigns a confidence bound to each quadrant to be updated in each round. Bounds decrease when a quadrant is more visited than other quadrants. The algorithm starts by exploring all the quadrants with the highest confidence bounds, finding the best quadrants after some exploration, and then reaps the benefits to maximize the profits.

### Mathematical model

More formally, the problem to be solved is as follows. Suppose we repeatedly interact with an environment characterized by the realization of certain spatial events (i.e., crime events). Spatial events are modelled as count random variables *X*_*i*,*t*_, where *i* indexes a spatial location and *t* indexes the round of the interaction. In each round we are given a chance to observe a finite non exhaustive number of locations (a subset *S* of all locations) and record the realization of these random variables (i.e., police can only visit a finite non exhaustive number of locations in the city). For those events that we did not observe in a particular round, we observe a filtered observation. That is, for each *i* ∉ *S*, we observe a count random variable ${\tilde{X}}_{i,t}$ (i.e., an underreported number of crime events). Our main hypothesis is that the count random variable ${\tilde{X}}_{i,t}$ is an underreported realization of the count variable *X*_*i*,*t*_. To fix ideas the reader can think *i* denoting a location in a city, *t* a date, *X*_*i*,*t*_ the number of crimes that occur at this location on a particular date, *S* as those locations that on date *t* are visited by police officers and ${\tilde{X}}_{i,t}$ the reported crime incidents of those places not visited by the police on that particular date but still reported by, for example, some citizens. Our objective is, in a repeated interaction with this environment, to learn the true mean of the distributions of spatio-temporal events *X*_*i*,*t*_ and filtered (or underreported) spatio-temporal events ${\tilde{X}}_{i,t}$. To formally set up the problem to be solved, we use the same notation as in [30], and rewrite [29, 31] algorithms in this notation.

The CMAB problem with *underreporting* consists of *M* *base arms* associated with a set of random variables *X*_*i*,*t*_ (i.e., crime events) and ${\tilde{X}}_{i,t}$ (i.e., underreported crime events), with bounded support in [0, 1], for 1 ≤ *i* ≤ *M* and *t* ≥ 1. Variables *X*_*i*,*t*_ indicate the random outcome of the *i*-th base arm in the *t*-th trial. Variables ${\tilde{X}}_{i,t}$ indicate underreporting of events *X*_*i*,*t*_. We assume that the set of variables {*X*_*i*,*t*_∣*t* ≥ 1} associated with base arm *i*, are independent and identically distributed over time *t* according to some distribution with unknown expectation *μ*_*i*_. We also assume that the set of variables $\left\{{\tilde{X}}_{i,t}|t\geq 1\right\}$ associated with underreporting of base arm *i* over time *t*, are independent and identically distributed according to some distribution with unknown parameters *q*_*i*_. Note that *X*_*i*,*t*_ and ${\tilde{X}}_{i,t}$ may be correlated.

Let ***μ*** = (*μ*_1_, *μ*_2_, …, *μ*_*M*_) be the vector of expectations of all base arms, and ***q*** = (*q*_1_, *q*_2_, …, *q*_*M*_) be the vector of the parameters of interest of the underreported base arms. Note that *q*, in our model, is not the mean of the vector ${\tilde{X}}_{t}$. By allowing random variables of different base arms to be dependent we rationalize the common framework in which the random variables {*X*_*i*,*t*_ ∣ *i* = 1, …*M*} represent the spatial events at *M* different locations. We also allow for the dependence of base arms and underreporting of base arms in the same period and across arms, as noted earlier.

In every period a decison maker or social planner (i.e., police planning department) must select a *super arm* (i.e., set of locations to be visited by police officers), which is a subset of the set of base arms. Let S denote the set of all possible super arms that can be played in a CMAB problem instance. For example, S can be the set of all subsets of base arms containing *m* base arms (this is our case). In each round, one of the super arms S∈S is selected and played, and every base arm *i* ∈ *S* is triggered and played as a result (i.e., this means that the realization of crime, *X*_*i*,*t*_ is observed). Therefore, the model relies on the strong assumption that the police can get to know all crimes in areas they visit each day. In practice, this assumption could be valid for some particular types of crime, for instance, in public spaces, such as crimes against public order, property crime, traffic offenses, and some kinds of violent crimes and property crimes, to which police may have access in small vigilance areas [32]. In addition, other information sources may also help to know about crimes, such as police intelligence which may provide crime information in the field [33], surveillance cameras [34], and geospatial data provided by social networks [35]. We assume also that for arms outside super arm *S*, we observe underreported realizations of the base arms. More precisely, we assume that for *i* ∉ *S*, we observe the random variables $\left\{{\tilde{X}}_{i,t}|X_{i,t}\right\}$ (i.e., the realization of underreporting conditional to the true crime realization). That is, arms not in the super arm selected in some rounds, are fired but not observed and we only observe the random variable ${\tilde{X}}_{i,t}$ conditional to *X*_*i*,*t*_. In our simulation study and applications we assume that variables *X*_*i*,*t*_ distribute as a Binomial random variable *B*(*n*, *μ*_*i*_), and ${\tilde{X}}_{i,t}$ conditional to *X*_*i*,*t*_, which we denote as $\left\{{\tilde{X}}_{i,t}|X_{i,t}\right\}$, are distributed as a Binomial random variable with parameters *X*_*i*,*t*_ and *q*_*i*_, denoted by *B*(*X*_*i*,*t*_, *q*_*i*_).

For each arm *i* ∈ {1, …, *M*}, where *M* is the total number of arms, let *T*_*i*_(*t*) denote the number of times arm *i* has been triggered after the first *t* rounds in which *t* super arms have been played. If arm *i* ∈ *S* is not triggered in round *t* when super arm *S* is played, then *T*_*i*,*t*_ = *T*_*i*,*t*−1_. Analogously, let ${\tilde{T}}_{i}\left(t\right)$ denote the number of times arm *i* has been underreported after the first *t* rounds in which *t* super arms have been played.

The final reward of a round depends on the outcomes of all triggered base arms in the super arm. Let *R*_*t*_(*S*) be a non-negative random variable denoting the reward of round *t* when super arm *S* is played. We assume that reward *R*_*t*_(*S*) has the form $R_{t}\left(S\right)=\sum_{i\in S}X_{i,T_{i,t}}$. In other words, our goal was to maximize the number of observed incidents. Underreported events do not contribute to the reward. The expected value of *R*_*t*_(*S*), *E*[*R*_*t*_(*S*)], is a function of *S* and the parameters *μ*_*i*_ of the arms in super arm *S*.

An algorithm for this problem is the selection of a super arm for each round *t* such that it maximizes the expected round *t* reward: *E*[*R*_*t*_(*S*)] = ∑_*i*∈*S*_*μ*_*i*_, for an unknown ***μ***. To use the algorithms proposed by [29–31], we must have access to a computational oracle that takes an expectation vector *μ* as input, and compute the optimal or near-optimal super arm *S*. In our case, the computational oracle is reduced to a sorting problem for which there are fast algorithms [36].

### Algorithms

For completeness and to illustrate how we apply the algorithms [29–31] to our underreporting problem, we provide the pseudo-algorithms that we implemented.

**Algorithm 1 Combinatorial Upper Confidence Bound Algorithm (CUCB) with underreporting.**

1: For each arm *i*, maintain: (1) variable *T*_*i*_ as the total number of times arm *i* is played so far; (2) variable ${\tilde{T}}_{i}$ as the total number of times arm *i* has been underreported (initially both 0); (3) variables ${\hat{\mu }}_{i}$, ${\hat{q}}_{i}$ as the mean of all outcomes *X*_*i*,*t*_ for 1 ≤ *i* ≤ *M* that have been observed up to round *t* and the best estimate of the parameters characterizing ${\tilde{X}}_{i,t}$, 1 ≤ *i* ≤ *M*, which have been observed up to round *t* (initially both 1).

2: *t* ← 0.

3: **while true do**

4:  *t* ← *t* + 1.

5:  For each arm *i*, set ${\bar{\mu }}_{i}=\text{min}\{{\hat{\mu }}_{i}+\sqrt{\frac{3\text{ln}t}{2T_{i}}},1\}$

6:  $S=\text{Oracle}({\bar{\mu }}_{1},{\bar{\mu }}_{2},\ldots ,{\bar{\mu }}_{m})$.

7:  Play *S*. Observe the outcomes of base arms *i* ∈ *S*, and update all *T*_*i*_’s and ${\hat{\mu }}_{i}$’s.

8:  For *i* ∉ *S*, observe ${\tilde{X}}_{i,t}$ conditional on the outcomes of base arm *i* in step 7. Update ${\hat{q}}_{i}$:

$$\begin{array}{c} {\hat{q}}_{i}\leftarrow \frac{\text{Empirical}\;\text{mean}\;\text{of}\;\text{underreporting}\;\text{so}\;\text{far}\;\text{observed}}{n{\hat{\mu }}_{i}} \end{array}$$ (1)

9: **end while**

With this notation we write the **Learning with Linear Rewards (LLR) algorithm** of [31] as follows. Replace Step 5 in 1 with:

$$\begin{array}{c} \bar{\mu }={\hat{\mu }}_{i}+\sqrt{\frac{(M+1)\text{ln}t}{T_{i}}} \end{array}$$ (2)

Finally, we consider the **CUCB, version 1 (UCB1) algorithm**, of [29] which ignores the potential association between arms at any moment in time. This is a major handicap in its performance as has been pointed out in [31]. Replace Step 5 in 1 as follows. Rather than choosing a super arm every time period, the algorithm updates only the arm that maximizes:

$$\begin{array}{c} {\hat{\mu }}_{i}+\sqrt{2\frac{\text{ln}t}{T_{i}}} \end{array}$$ (3)

### Estimating crime underreporting

**Fig 2:**
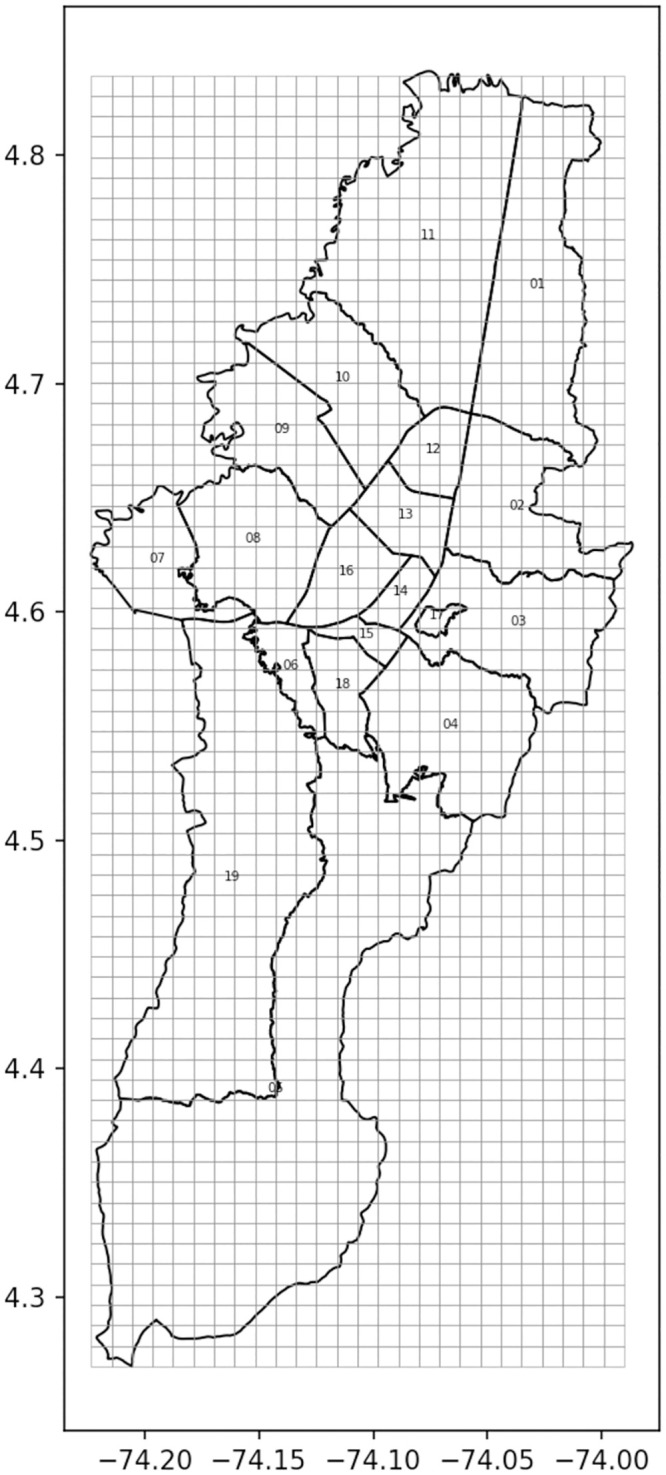
Bogotá, capital city of Colombia. Figure shows the 19 jurisdictions in which the city is divided and our grid of 1 km^2^ cells. This figure was created by the authors using a shapefile of the administrative division of Bogotá, which is publicly available on the government’s “Datos abiertos” (Open data in Spanish) web page at https://datosabiertos.bogota.gov.co/dataset/localidad-bogota-d-c.

We provide two applications of our underreporting algorithm, showing that it is an effective way of estimating the true mean of crime incidents and underreporting in Bogotá, the capital city of Colombia. First we discuss how we built the two data sets for our applications. The first was the real crime and underreporting dataset and the second was, the simulated dataset. We divided the city into 1 km^2^ (1 *km* × 1*km*) cells. This resulted in 500 cells with at least one crime during year 2018. These cells were the focus of our study. In both applications we assumed that the size of the superarms was at most 10% of the number of arms. This is because the number of arms that can be efficiently monitored and spot checked by police officers is at most 10% of the area of the city’s area. Note that according to official statistics [37], between years 2012–2015, all homicides and 25% of crime in the city took place in 2% of street segments. Fig 2 shows the 19 jurisdictions in which the city is divided and our grid of 1 km^2^ cells that we used as arms.

### Crime data

Our dataset contained daily time-stamped information on the spatial location of each criminal event reported in Bogotá from January 2018 to December 2019. The source was the Criminal, Contraventional and Operating Information System (SIEDCO). The dataset was assembled by the Colombian National Police and was provided by the Bogotá Security Office. Although SIEDCO is the official crime source in the city, there is evidence of substantial underreporting as can be deduced from two different sources. The first source is citizens crime reports to the security and emergency call center NUSE (*Número Único de Seguridad y Emergencias* in Spanish). By comparing the different reports in SIEDCO and NUSE, it can be observed that many reports in NUSE do not appear in SIEDCO and viceversa.

**Fig 3:**
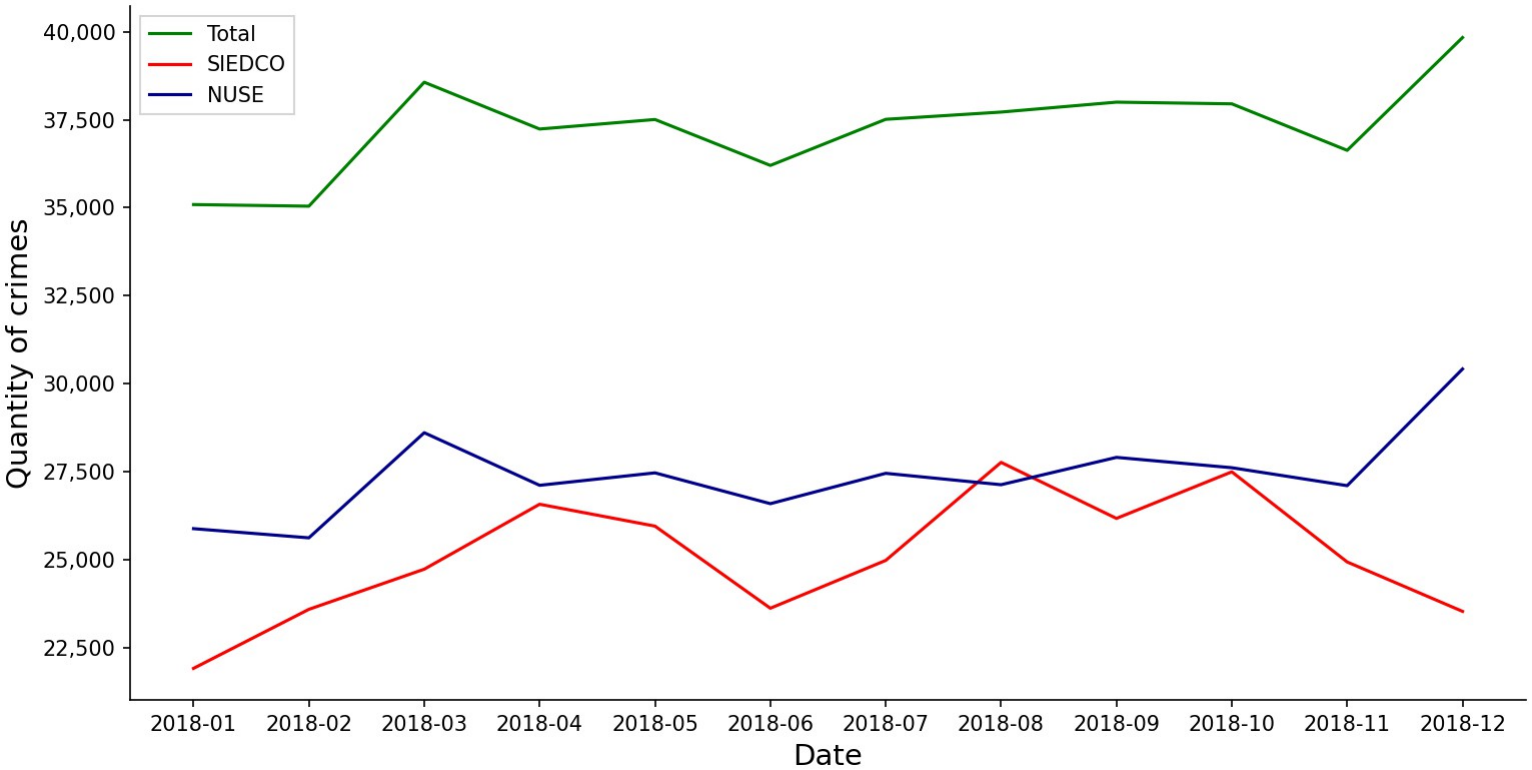
Crimes by source of information: SIEDCO is the official source of information of crimes in Bogotá. NUSE is the security and emergency call center of the city. Total is the sum of both sources eliminating double counting as explained in the main body of the text.

Our main approach for capturing the totality of violent crimes consists of combining both data sets. To avoid double counting of crimes, we eliminated all crimes *a* for which there was another crime *b* that belonged to the same crime category, occurred at a distance of less than 500 meters and both where reported within a period of less than 8 hours. Fig 3 shows the total number of crimes reported by each source, SIEDCO and NUSE, and the Total number of crimes which is the sum of SIEDCO plus NUSE eliminating double counting as explained previously. This Total series (the green line in Fig 3) is called the *real* dataset.

The second source is Bogotá’s City Chamber of Commerce (*Cámara de Comercio de Bogotá*, in Spanish) victimization and reporting survey [38]. This is a biannual crime perception and victimization survey that asks individuals if they had been victims of some crime in the last six months and in case they did, if they had reported this crime. The survey is representative of the whole city, stratified at the level of 19 jurisdictions of Bogotá. The universe of the survey was all citizens over 18 years of age in the city of Bogota, inhabitants of the 19 urban localities of the city and belonging to all economic classes. The survey is carried out by telephone and has a sample size of 9,527 people. The sampling was stratified and multistage random, representative at the city level. The degree of confidence is 95% and the margin of error is 2.7%. In 2021, the survey reported an average victimization rate of 17% and, among those, only 27% said they had reported the event to the police. Using this survey, we simulated a second dataset using standard crime models. To construct the second dataset, we first fit a Poisson model at the cell level for all crimes in the first dataset. This model simulated the crimes for each round of the algorithms. The underreported data was computed using the reporting rate from Table 1. In particular, cells were mapped to a specific jurisdiction by considering the jurisdiction containing its centroid. Following this, the reporting rate per jurisdiction was multiplied by the number of crimes provided by the Poisson model to estimate the underreported crimes at each cell. No additional information was used. The central assumption of this estimation was that the survey captures the number of underreported crimes well. Therefore, we ignore possible heterogeneities in underreporting among cells for the same jurisdiction. Importantly, since this survey is done every six months at a considerable cost, one of our contributions is to provide a methodology that estimates underreporting rates with the same frequency in which crime data is collected in the city, i.e., daily.

**Table 1:** Results of Bogotá’s City Chamber of Commerce, Cámara de Comercio de Bogotá, victimization and reporting survey 2014. We use reported rates form each jurisdiction to estimate underreporting simulated from our Poisson model. The table also reports the population of each jurisdiction and victimization rate.

ID	District	Pop.	Vict. Rate	Rep. Rate
15	Antonio Nariño	109,176	15%	33%
12	Barrios Unidos	243,465	12%	22%
07	Bosa	673,077	13%	26%
17	Candelaria	24,088	12%	22%
02	Chapinero	139,701	9%	28%
19	Ciudad Bolívar	707,569	8%	17%
10	Engativá	88,708	11%	20%
09	Fontibón	394,648	10%	19%
08	Kennedy	1,088,443	13%	28%
14	Los Mártires	99,119	17%	25%
16	Puente Aranda	258,287	14%	32%
18	Rafael Uribe Uribe	374,246	12%	15%
04	San Cristóbal	404,697	13%	21%
03	Santa Fe	110,048	17%	17%
11	Suba	1,218,513	5%	19%
13	Teusaquillo	1,53,025	14%	19%
06	Tunjuelito	19,943	17%	23%
01	Usaquén	501,999	18%	13%
05	Usme	457,302	9%	33%

## Results

This work proposed a model for recovering “true” crime and underreported incident rates using a combinatorial multi-armed bandit framework. First, we present results related to the validation of the proposed strategy. Then, we report results related to the estimation of crime using emergency reports. Finally, we report the results of crime estimations from simulations based on citizen survey data.

### Model validation

To validate our strategy to elicit the “true” incidence rate, underreporting parameters, and maximize the discovered events simultaneously, we extensively study the model with binomial arms distributions and binomial conditional underreporting. We report the results of the four experiments. In all of our validation simulations and in our two applications, we assume that the size of the super arms is at most 10% of the number of arms. This is because in our applications to crime underreporting, the number of arms that can be efficiently monitored and checked by police officers are at most 10% of the area of the city.

**Fig 4:**
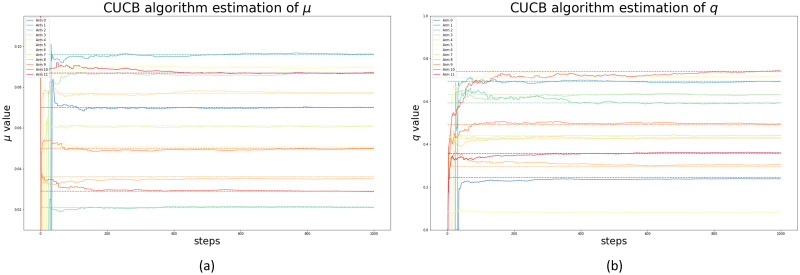
CUCB Convergence. Panel (a), CUCB Convergence to true arms mean. Panel (b), CUCB Convergence to true arms underreporting parameters.

In the first experiment we used 12 arms and considered the superarms of at most two arms as shown in Table 2. The true mean ***μ*** and parameters ***q*** for the first set of simulations are listed in Table 3. Fig 4 at Panel (a) and (b) show how the CUCB algorithm converges to the true values of *μ* and *q* over time for the different arms, which are represented by dashed horizontal lines in both figures. The graphs for UCB1 and LLR are similar and are not shown for brevity.

**Table 2:** Global parameters. *M* is the number of arms, *m* the size of the super arm, *T*_*max*_ the maximum number rounds played and *n* is the parameter of the Binomial distribution.

*M*	*m*	*T*_*max*_	*n*
12	2	1000	1000

**Table 3:** True values of *μ* and *q* for each arm in simulations.

Arm	*μ*	*q*
0	0.070	0.244759
1	0.096	0.694755
2	0.021	0.593902
3	0.087	0.631792
4	0.061	0.440257
5	0.090	0.083726
6	0.051	0.712330
7	0.077	0.427863
8	0.036	0.297780
9	0.050	0.492085
10	0.029	0.740296
11	0.087	0.357729

**Fig 5:**
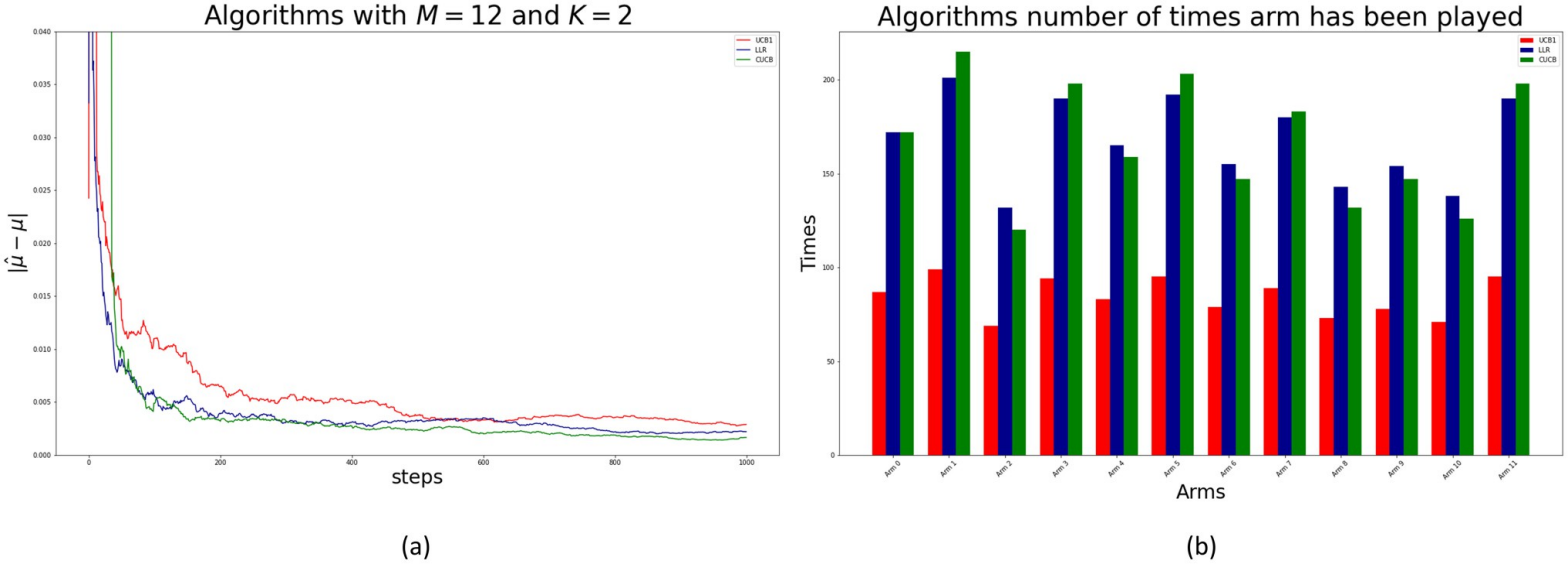
Algorithms convergence error and number of visits. Panel (a), convergence error of true arms mean for each algorithm. The error is measured as the Euclidean distance between the true mean vector and the estimated mean vector per round. Panel (b), number of visits (i.e., fired arms) of algorithms to each arm.

Fig 5 at Panel (a) shows the Euclidean distance between the estimated ${\hat{\mu }}_{t}$ and true value of *μ* in each round *t* of the algorithms. Additionally, Panel (b) shows the number of times each algorithm triggered each arm. Note that, by construction, UCB1 visits only one arm per round while the other two algorithms visit all arms in the superarm in each round. Hence, after 1.000 rounds, the other algorithms visited mores arms. Note also that there were minor differences in the number of times each arm is visited by CUCB and LLR algorithms. Finally, given that this was a small simulation experiment, there is no major computational burden. The results clearly show that all algorithms in this small experiment can recover the true means of all arms and the true parameters of the underreporting distributions. Fig 5 shows how the CUCB algorithm (green line) outperforms the other two algorithms in terms of convergence speed.

**Fig 6:**
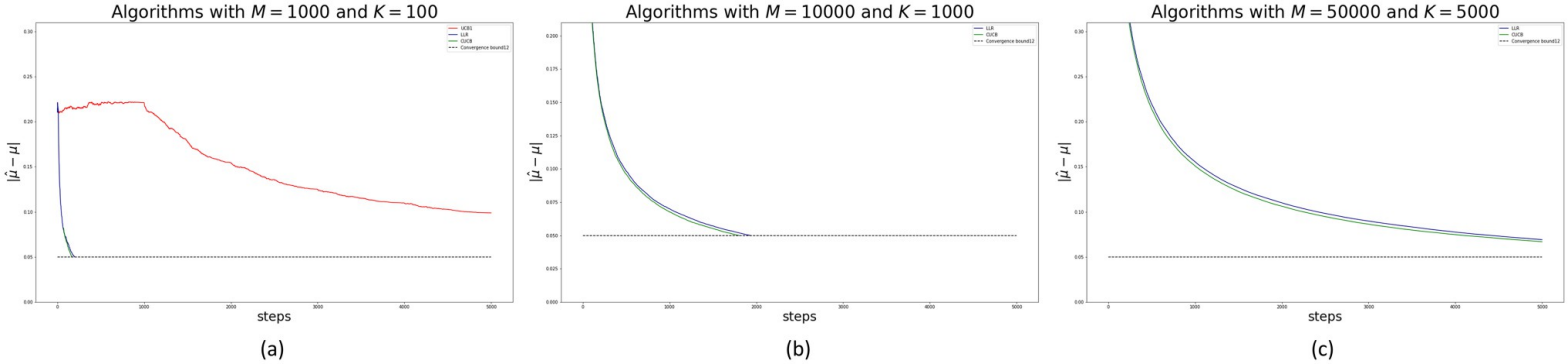
Convergence error of true arms mean for each algorithm. The error is measured as the Euclidean distance between the true mean vector and estimated mean vector per round.

The next experiment solved an increasingly challenging task. In each arm, we drew random true mean incidence rates, ***μ*** and parameters, ***q*** for each arm. Fig 6 at Panel (a) shows the case of 1.000 arms and at most 100 super arms. Since UCB1’s performance is highly surpassed by the CUCB and LLR algorithms, we do not report the outcome of this algorithm in the next two exercises. Fig 6 at Panel (b) and Panel (c) report the cases of 10.000 and 50.000 arms with at most 1.000 and 5.000 super arms, respectively. These figures show on the Euclidean distance between the true mean and the estimated values in each round the vertical axis. In addition, Table 4 reports the time required for each algorithm to complete 1.000 rounds (we used a portable PC, with Intel i7-16 GB of RAM).

**Table 4:** Time to completion of 1, 000 rounds of each of the three algorithms. Case 1: *M* = 1, 000 and *K* = 100. Case 2: *M* = 10, 000 and *K* = 1, 000. Case 3: *M* = 50, 000 and *K* = 5, 000. Sec is seconds, min is minutes.

	Case 1	Case 2	Case 3
UCB1	3 sec	38 sec	3 min 31 sec
LLR	4 sec	51 sec	4 min 15 sec
CUCB	4 sec	53 sec	4 min 12 sec

Table 4 quantifies the time to completion of 1.000 rounds for each algorithm. With many arms, CUCB and LLR have a similar performance, but after 1.000 rounds UCB1 fails to converge.

### Underreporting of crime using emergency reports

**Fig 7:**
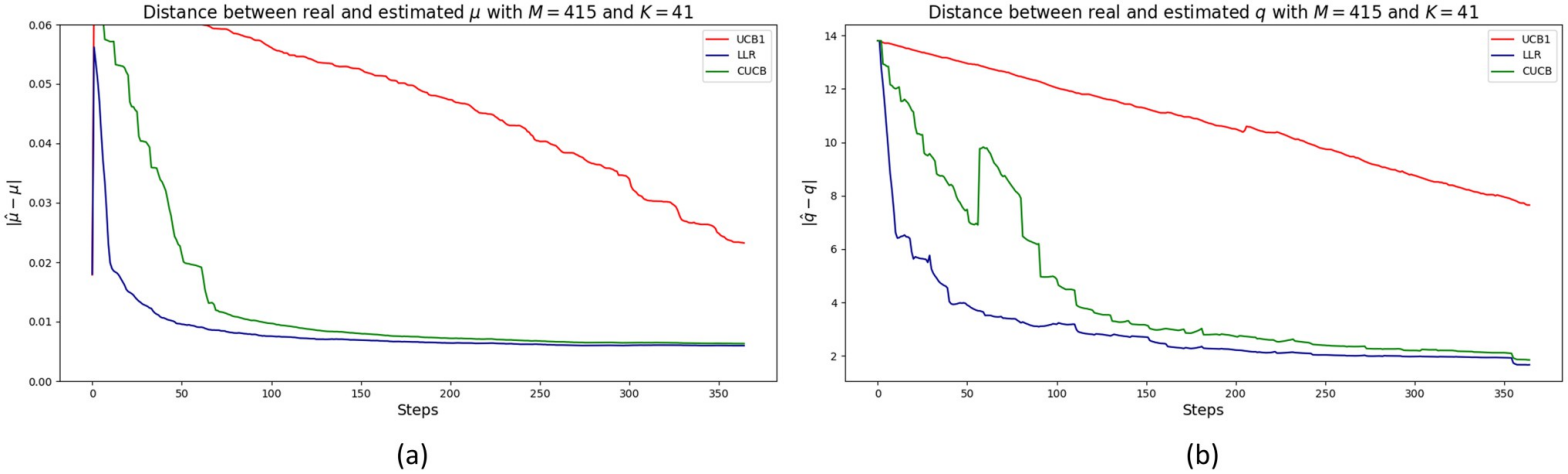
Panel (a), convergence of the vector of incidence rates ***μ*** to the mean of all crimes per cell across time. The error is measured as the Euclidean distance between vectors with 415 components. Panel (b), convergence of estimated vector ***q*** per round to the empirical mean of the underreporting rate for the whole sample. The error is measured as the Euclidean distance between vectors with 415 components.

Consider our first application in which we have done our best to estimate the real crime rate and underreporting in each cell of Bogotá in 2018 (what we call the real dataset). Below we present the results of running the three algorithms on these datasets. Fig 7 at Panel (a) and (b) show the convergence of the vector of incidence rates ***μ*** and the vector of parameters ***q*** respectively, for each algorithm. In each case the reference vectors are the mean of all crimes in each cell over the period and the mean of the vector of estimated underreporting parameters in each cell over the entire period. In either case, the error is measured as the Euclidean distance between two high dimensional vectors with 415 components. Therefore, a reported error of for example, 0.4 in Fig 7 at Panel (a), or 2 in Panel (b) represents means errors per component of 0.96^−3^ and 4.8^−3^ respectively. In addition, these parameters are unknown in this real-world application.

As expected, the estimated parameters is not perfect because the real dataset may not satisfy some of our working hypothesis. In particular, the number of crimes reported per cell *i* as a proportion of the total number of crimes in the cell, $\frac{\tilde{X_{i}}}{X_{i}}$, may not be a stationary distribution. In addition, the distribution of $\tilde{X_{i}}|X_{i}$ may not be a binomial random variable, *B*(*X*_*i*_,*q*_*i*_). Note that since many cells report zero crime, care must be taken to empirically estimate these ratios. To do these, we estimate the mean $\tilde{X_{i}}|X_{i}$ whenever *X*_*i*_ ≠ 0, otherwise we set the ratio to zero. We compare these statistics to those implied by the model: *q*_*i*_(1 − (1 − *μ*_*i*_)^*n*^).

**Fig 8:**
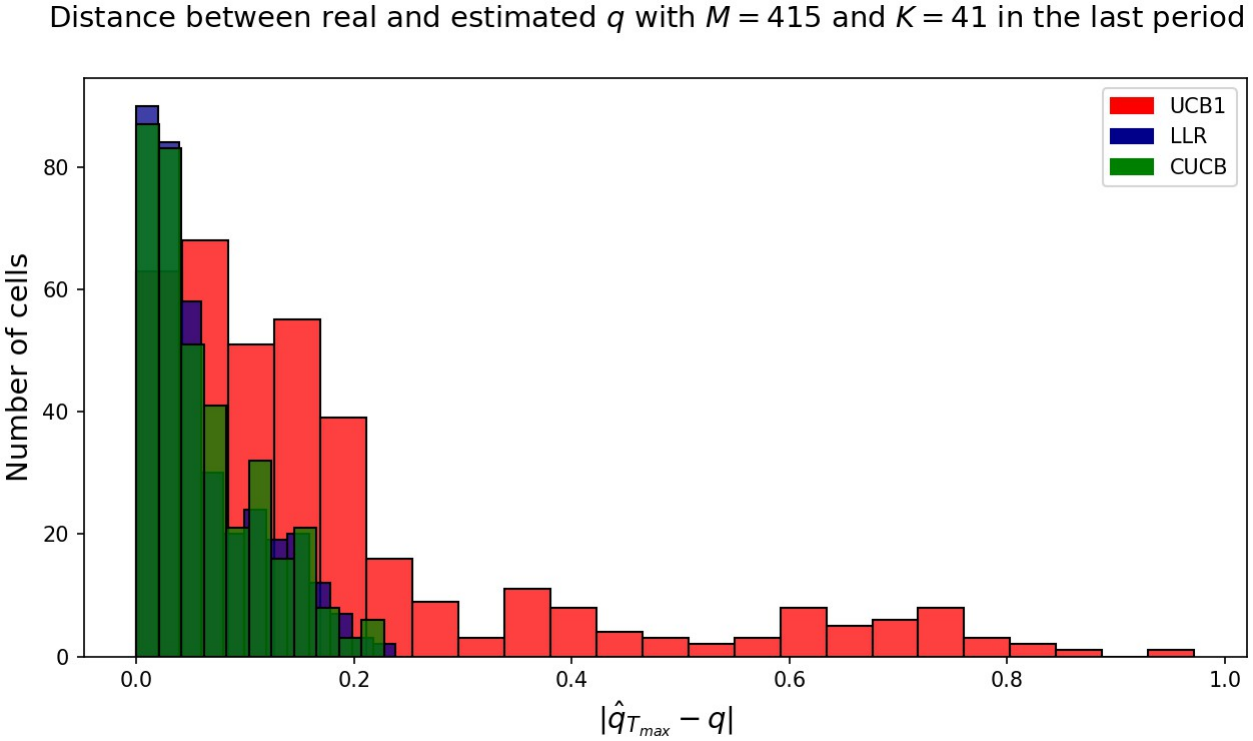
Histogram of convergence of estimated error of *q* in the last round to the empirical mean of the underreporting rate for the whole sample. Absolute values reported.

We further explore the nature of this convergence. Fig 8 shows a histogram of the error between the empirical mean of the ratio $\frac{\tilde{X_{i}}}{X_{i}}$ and that implied by our model in the last round per cell (error in absolute value). As can be seen from Fig 8, the CUCB and LLR algorithms converge in almost all cells with an error smaller than 0.2, after 1, 000 rounds.

**Fig 9:**
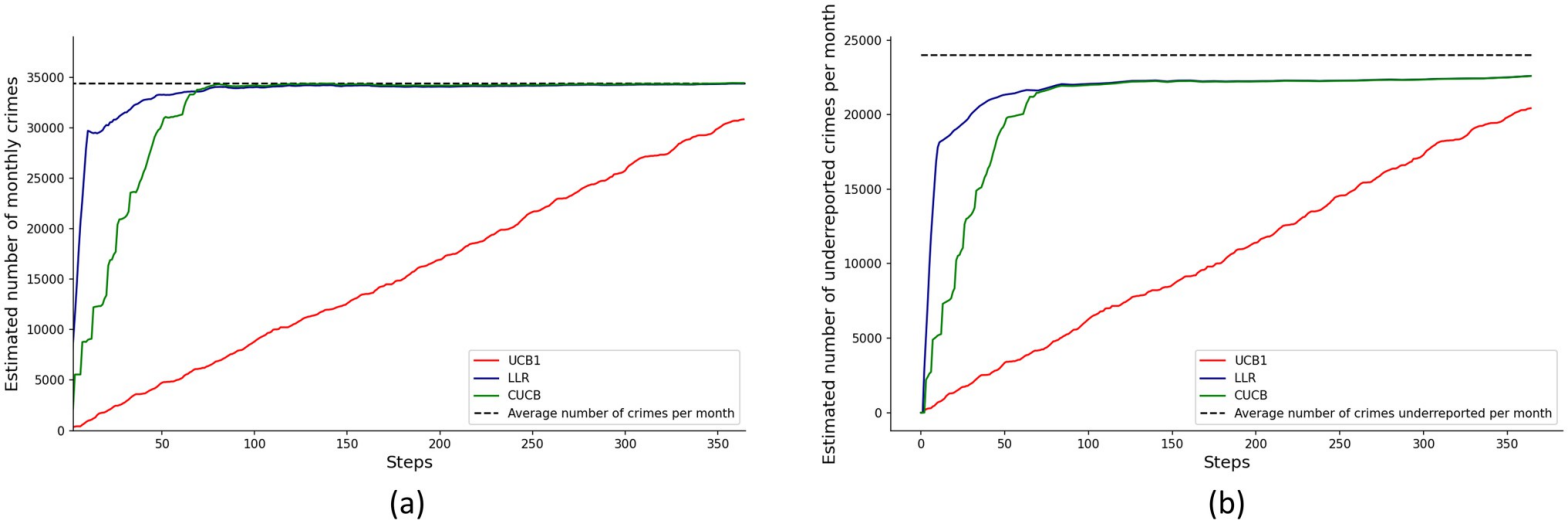
Panel (a), convergence of the estimated total number of crimes to the observed number of crimes in the city. Panel (b), convergence of the estimated total (aggregate across cells) of the number of underreported crimes implied by the model.

Fig 9 at Panel (a) and Panel (b) show the model implications for aggregate crime and underreporting (compare to Fig 3). Specifically, Fig 9(b) shows the aggregate expected crime rate over all cells in each round, *n*(*μ*_1_ + …+ *μ*_415_). Note that the CUCB and LLR algorithms converge approximately to the most recent observation of Total in Fig 3. In addition, Fig 9 at Panel (b) shows the expected value of total underreporting in each round: *n*(*μ*_1_*q*_1_ + …+ *μ*_415_*q*_415_). The CUCB and LLR algorithms converged approximately to the most recent NUSE observations. However, as noted before, the convergence of the vector of parameters ***q*** is not equally good across all cells, and hence there is an aggregate discrepancy.

**Fig 10:**
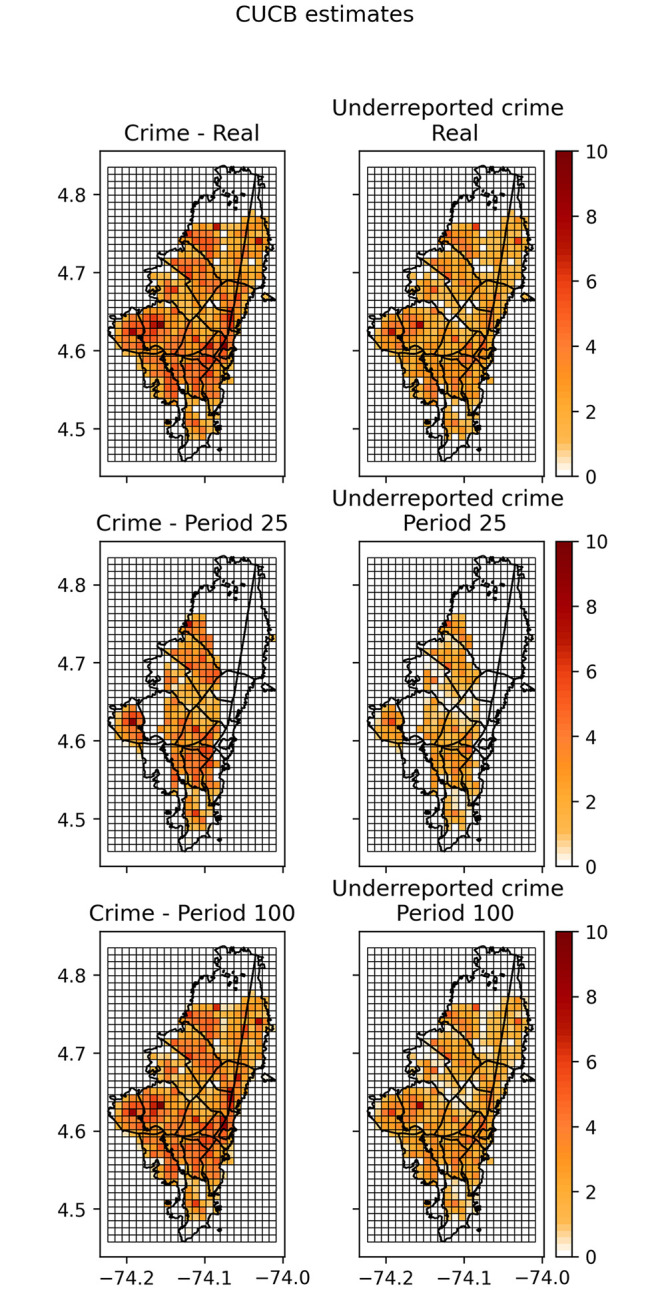
Heat map illustrating the convergence, using the CUCB algorithm, of the estimated crime and underreporting of events in the city, to the real values. The first column, second and third rows show the heat maps of the estimated crime incidence rates after 25 and 100 iterations, respectively. The second column, first row shows real underreporting as measured by NUSE dataset. The second column, second and third rows show the heat maps of the estimated underreporting crime after 25 iterations and 100 iterations, respectively. This figure was created by the authors using a shapefile of the administrative division of Bogotá, which is publicly available on the government’s “Datos abiertos” (Open data in Spanish) web page at https://datosabiertos.bogota.gov.co/dataset/localidad-bogota-d-c.

A more illustrative presentation of the results is shown in Fig 10. We only report the results for the CUCB algorithm. The first column and row of the panel in Fig 10 show a heat map of the estimated real crime incident rates in the city and how the CUCB algorithm discovered these crime incidents. The first, second and third rows (left column), show the heat maps of the estimated crime incidence rates after 25 iterations and 100 iterations of CUCB, respectively. The first row, second column, show real underreporting as measured by NUSE dataset. The second and third rows (second column), show the heat map of the estimated underreporting crime after 25 and 100 iterations of CUCB, respectively.

### Underreporting of crime using survey based data

**Fig 11:**
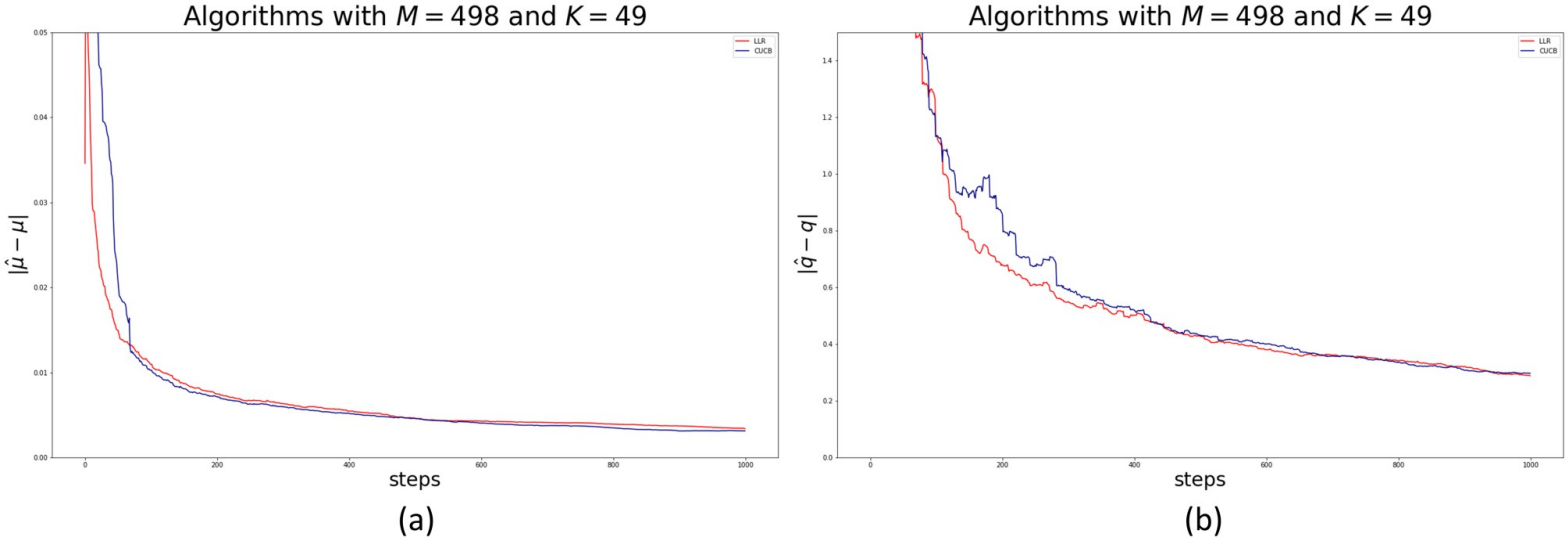
Panel (a), results for second application simulating data with standard crime Poisson model. Panel shows the convergence of the vector true incidence rates *μ* to the true values. Error measured as Euclidean distance between vectors. Panel (b), results for second application simulating data with a standard crime Poisson model. Figure shows the convergence of the vector parameters ***q*** to the true values. Error measured as the Euclidean distance between vectors. UCB1 not reported because it is outperformed by the other two algorithms.

In our second application, we estimated a standard crime model. Using historical data, we fitted a Poisson distribution to each cell and used the Bogotá’s City Chamber of Commerce 2014 victimization and reporting survey to estimate underreporting in each cell (note that the underreporting rate is the same for all cells that are mapped to the same jurisdiction). Fig 11 at Panel (a) shows the convergence of the vector of the true incidence rates ***μ*** to the true values. The error was measured as the Euclidean distance between the vectors. Note that the UCB1 algorithm failed to converge after 1.000 rounds.

Finally, in Fig 11 at Panel (b) we report the convergence of the vector of parameters ***q*** in the underreporting distribution. The error was measured as the Euclidean distance to the true parameters. Algorithm UCB1 is not shown because it was considerably outperformed by the other two algorithms.

## Discussion

This paper studies the “true” crime incident rates estimated over time using data from underreported crime observations and complementary crime-related measurements acquired incrementally. Two crime-related observational mechanisms exhibiting underreporting, namely, official crime registers and victimization surveys, were studied to estimate their underreporting and “true” incidence rates, unveiling their dark crime figures over time. In contrast to previous approaches for estimating crime underreporting, which mainly focused on the long-term adjustment of underreporting rates, this study describes for the first time the online estimation of the spatial crime rates by sequentially integrating time-varying complementary crime-related observations.

The underreporting of spatio-temporal events is ubiquitous in many social problems [39], and particularly for crime characterization [8, 40]. All systems that describe crime dynamics, including official crime registers and citizen surveys, provide informative but limited observations of crime occurrences [40]. Concerns about the dark crime figure have been present since the first initiatives to study crime quantitatively [41] until the modern artificial intelligence strategies for crime prediction [19]. Underreporting is present not only in the spatial dimension but also in the temporal dimension [42]. Previous works on crime underreporting focused on constructing average crime rate estimations for long-term windows [6, 9, 17, 18, 27]. Most of these works aim to quantify the spatial underreporting of official crime registers, assuming the citizen’s surveys on victimization as the crime ground truth. This work also provides similar spatial estimations of underreporting but accounts for the temporal dimension, providing spatiotemporal estimates of the “true” crime incidences. Previous work on crime prediction also considers a time-based crime characterization but does not account for the underreporting phenomena [1, 43, 44]. In a recent paper, Brunton-Smith et al. [21] have pointed out an oversimplifying assumption conventionally used for estimating the underreporting of crime events: the undercounting of events being independent of any other area characteristics or uniform across geographic scales. Although we partially rely on this assumption in the survey-based application, we allow for non-uniform variation in underreporting rates across jurisdictions but not within the cells in each jurisdiction. Our model accounts for any dependence between cells (geographical areas). The only, although strong, assumption we make is that crime and underreported events are independent across time. Extending the proposed model to this case should account for time dependence which may require a complete Markov decision process [45], which can be explored in future research. Our results show that combining complementary crime-related data sources over time may help gradually illuminate the dark figure of crime, as illustrated Fig 10. We would also like to underscore that the paper’s main point is not to put forward the idea that the two datasets we constructed to validate our methodologies are faithful representations of the real crime and underreporting of crimes in the city. Instead, we use these as plausible examples of real crimes and underreporting rates and show that in these examples, our algorithm is capable, by repeatedly interacting with the environment, of identifying the true crime and underreporting rates. Moreover, the technique may be used to have daily estimates of these data.

The proposed approach estimates the “true” crime incidence over time using synthetically constructed ground truths of crime. It is worth noting that constructing “real” crime reference databases is a challenging problem, mainly because it is almost impossible to directly measure this phenomena [8]. Our experimental configuration relies on two settings that explore the proposed approach capabilities to discover underreporting on two simulated ground truth crime databases. The first setting aimed to investigate the capacity of the proposed approach to complement official crime reports, with information supplied by citizens. Previous works suggest that official crime reports are biased by underreporting [3–6]. This limitation may result from unequal reporting rates across the population and space. The explored setting aimed to cover, at least partially, this reporting gap by considering, in addition, complementary reports, particularly citizens’ telephone calls crime-related reports [23, 46]. Therefore, a first ground truth crime database was constructed by combining these two datasets. Our results suggest that the proposed method provides good-quality ground truth crime estimations early in time (see Figs 7 and 11), even for estimations of the total number of crimes (see Fig 9). Nevertheless, the underreporting described for this setting should be cautiously interpreted because of the potential contamination of false crime reports, naturally observed in telephone reports of crime incidents [23, 46], which may result in over/sub estimation of the underreporting and “real” crime rates. The second setting aimed to overcome this limitation by considering a citizen victimization survey, which also accounts explicitly for underreporting [38]. Crime occurrences with underreporting were simulated in time and compared with ground truth reports of crime obtained from the same survey. Our results show that again in this alternative setting, the proposed method resulted in fast, good-quality estimations of crime underreporting, see Fig 11. However, these results should also be carefully interpreted because surveys provide long-term average descriptions of crime events, and the simulation process considered does not account for particular crime dynamics in time.

This work introduces a novel underreporting model of spatiotemporal events. The model relies on the multi-armed bandit framework, which provides efficient algorithms and convergence guarantees for online learning of the mean of the true arms distributions. Three well-known multi-armed bandit algorithms [29–31] were explored for the online estimation task. Importantly, the capacity of the model was extensively studied in several controlled simulated scenarios, given highly competitive results, as shown in Fig 4 and Table 4. Results in the crime underreporting estimation suggest the CUCB algorithm’s effectiveness in identifying the proposed model’s fundamental parameters. Furthermore, these results indicate that the combinatorial nature of CUCB may help to accelerate the crime underreporting discovery process, likely improving its exploratory capacity [30], as shown in the comparison between the algorithms in Fig 8.

Several studies have pointed out the potential pitfalls of using discovered crime incidents, biased or underreported, to train machine learning models that will be used for crime prediction and police allocation [19, 47, 48]. Previous approaches used urns models to show how a naive online learning algorithm cannot succeed in estimating the true distribution of events when discovered events and reported events have different incidence rates, and there is a feedback loop between the discovered events and the instrument used to monitor locations [49]. However, implementing this model in a large multi-armed setting is computationally expensive. The approach proposed here, based on multi-armed bandit problems, is more computationally efficient.

This work has some limitations. First, the evidence we report relies on simulated data. An actual implementation of the strategy in operational settings requires a mechanism to acquire “true” observations of crime sequentially (e.g., that here we approximate by police visits). For underreported crime events we use telephone calls related to crime. Alternative observational mechanisms can be implemented by considering, for instance, the information provided by citizens using other channels beyond the telephone or the information provided to police in situ during street surveillance, among others. Moreover, crime observation through these mechanisms may be affected by the strategic response of criminals, which are not considered in this work [11, 50]. Future work may consider a closed-loop estimation of crime underreporting considering criminal adaptation [11, 51]. Second, regarding the construction of our second dataset, we made the strong assumption that the victimization survey measures crimes and under-reporting where crimes actually happen as opposed to the places where people reside. As pointed out by [15], survey-based offense location of crimes is a better approximation to police records. Unfortunately, we do not have a survey that asks for the offense location, but rather, we assume that the offense took place at least in the same jurisdiction where the person resides. This may be a crude approximation to offense location, but given the size of each jurisdiction, there are nineteen in the whole city, the approximation might not be very bad if, for example, most people work and are in the same jurisdiction where they reside. As pointed out before, our main goal is not to show that our estimates of crime and under-reporting are correct but that they are plausible ground truths and, in any of the two cases, our methodology is able to discover this ground truth. Third, one of the main limitations of our work is the assumption that the police perfectly observe all crimes in the places they visit. This assumption has been used previously in the literature (e.g. [49]) and is sufficient to identify the parameters of interest: the total number of crimes in a region. This assumption is implausible in reality, as it may be that even under heavy police presence, some crimes are not observed or that increased police surveillance discourages victims from reporting [52]. Thus, our work only estimates the number of *observable* crimes, i.e., those with a non-zero probability of being observed by the police. This corresponds to a lower bound on the total number of crimes. If the unobservable crime rate is low (as it may be for some violent crimes or crimes on public roads), our method provides a better bound than the ones inferred using police statistics or victimization surveys alone. Nevertheless, if the unobservable crime rates are spatially and temporally heterogeneous, the results of our methodology would still suffer from biases similar to those of the official statistics. If we were to avoid the assumption of perfect observability, then the total number of crimes would be unidentifiable from the observable data. Alternatively, we could assume parametric forms for the underreporting processes to appropriately estimate the total number of crimes. However, these assumptions seem more restrictive, and we leave the exploration of alternative identifying assumptions for future work.

As future work, the estimation of the “true” crime incident rates from official crime records could be informed by citizen’s victimization’s surveys, as recently explored for quantification of underreporting [6]. Finally, the estimated underreported crime rates correspond to the particular case of Bogotá (Colombia), a large Latin American city with a specific crime dynamic and citizen’s reporting habits. Further work may explore the possibility of computing these estimations for other cities where reporting and crime dynamics may change.

## Conclusions

This paper studied the estimation of “true” crime over time from underreported crime observations by sequentially considering complementary crime observations. For this, a novel multi-armed bandit model for underreporting estimation was proposed. Efficient algorithms for online learning of the mean of the true-crime distributions in different areas were studied and validated for identifying the fundamental model parameters. This strategy was applied for estimating crime underreporting on two data sources: official crime reports and citizens’ victimization surveys. In the first setting, an estimate of the “true” crime incidence rate per geographical unit (1 km^2^ cells) and crime underreporting (our true crime scenario) were computed, and underreporting rates were estimated. The second experiment used an estimated Poisson model of crime incidence to simulate real crimes and estimate underreporting using a victimization and reporting survey conducted by the Bogotá’s City Chamber of Commerce. In both cases, our method performs well and suggests that this approach can be used to estimate, in an online setup, the underreporting of events. These findings may have implications in public policy because underreporting socially sensitive events can undermine the credibility of official figures and can be strategically used by government agents or influential citizens.
